# Early neuroimaging and ultrastructural correlates of injury outcome after neonatal hypoxic-ischaemia

**DOI:** 10.1093/braincomms/fcab048

**Published:** 2021-03-26

**Authors:** Yu-Chieh Jill Kao, Seu-Hwa Chen, Chia-Feng Lu, Bao-Yu Hsieh, Cheng-Yu Chen, Ying-Chao Chang, Chao-Ching Huang

**Affiliations:** 1 Department of Biomedical Imaging and Radiological Sciences, National Yang Ming Chiao Tung University, Taipei 11221, Taiwan; 2 Department of Anatomy and Cell Biology, School of Medicine, College of Medicine, Taipei Medical University, Taipei 11031, Taiwan; 3 Department of Medical Imaging and Radiological Sciences, College of Medicine, Chang-Gung University, Taoyuan 33302, Taiwan; 4 Department of Medical Imaging and Intervention, Chang Gung Memorial Hospital, Linkou, Taoyuan 33305, Taiwan; 5 Department of Radiology, School of Medicine, College of Medicine, Taipei Medical University, Taipei 11031, Taiwan; 6 Department of Medical Imaging, Taipei Medical University Hospital, Taipei 11031, Taiwan; 7 Department of Pediatrics, Kaohsiung Chang Gung Memorial Hospital and Chang Gung University College of Medicine, Kaohsiung 83301, Taiwan; 8 Department of Pediatrics, National Cheng Kung University Hospital, College of Medicine, National Cheng Kung University, Tainan 70428, Taiwan; 9 Department of Pediatrics, College of Medicine, Taipei Medical University, Taipei 11031, Taiwan

**Keywords:** hypoxic-ischaemia, apparent diffusion coefficient, magnetic resonance imaging, transmission electric spectroscopy, rat pups

## Abstract

Hypoxic ischaemia encephalopathy is the major cause of brain injury in new-borns. However, to date, useful biomarkers which may be used to early predict neurodevelopmental impairment for proper commencement of hypothermia therapy is still lacking. This study aimed to determine whether the early neuroimaging characteristics and ultrastructural correlates were associated with different injury progressions and brain damage severity outcomes after neonatal hypoxic ischaemia. Longitudinal 7 T MRI was performed within 6 h, 24 h and 7 days after hypoxic ischaemia in rat pups. The brain damage outcome at 7 days post-hypoxic ischaemia assessed using histopathology and MRI were classified as mild, moderate and severe. We found there was a spectrum of different brain damage severity outcomes after the same duration of hypoxic ischaemia. The severity of brain damage determined using MRI correlated well with that assessed by histopathology. Quantitative MRI characteristics denoting water diffusivity in the tissue showed significant differences in the apparent diffusion coefficient deficit volume and deficit ratios within 6 h, at 24 h and 7 days after hypoxic ischaemia among the 3 different outcome groups. The susceptible brain areas to hypoxic ischaemia were revealed by the temporal changes in regional apparent diffusion coefficient values among three outcome groups. Within 6 h post-hypoxic ischaemia, a larger apparent diffusion coefficient deficit volume and deficit ratios and lower apparent diffusion coefficient values were highly associated with adverse brain damage outcome. In the apparent diffusion coefficient deficit areas detected early after hypoxic ischaemia which were highly associated with severe damage outcome, transmission electron microscopy revealed fragmented nuclei; swollen rough endoplasmic reticulum and degenerating mitochondria in the cortex and prominent myelin loss and axon detraction in the white matter. Taken together, different apparent diffusion coefficient patterns obtained early after hypoxic ischaemia are highly associated with different injury progression leading to different brain damage severity outcomes, suggesting the apparent diffusion coefficient characteristics may be applicable to early identify the high-risk neonates for hypothermia therapy.

## Graphical Abstract

**Graphical Abstract:**
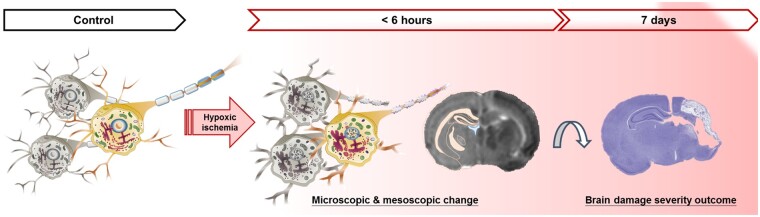

## Teaser

Kao et al. reported that apparent diffusion coefficient parameters depicted in early period after neonatal hypoxic ischaemia are highly associated with different brain injury outcomes. The changes in intracellular ultrastructure were observed within 6 h after hypoxic ischaemia and correlated with early apparent diffusion coefficient characteristics and final severity outcome.

## Introduction

Neonatal hypoxic ischaemia (HI) triggers cascades of neurotoxic events within hours that last for days to weeks after birth and evolve into HI encephalopathy.^1^^,^^2^ Neonatal HI brain injury may have serious neurological sequelae among survivors, including cerebral palsy, epilepsy, mental retardation and behavioural problems during long-term follow-up. Moderate hypothermia initiated within 6 h after delivery for infants with HI encephalopathy is now an evidence-based treatment.^3^

Hypothermia therapy decreases the risks of cerebral palsy and moderate or severe disability at school age in new-borns with moderate or severe HI encephalopathy.^4^^,^^5^ However, the progressive development of different severities of HI encephalopathy within a week after birth, namely mild, moderate and severe that correlate with long-term outcomes, cannot be reliably predicted clinically within hours after birth.^6^^,^^7^ Even though the neonate may display transient improvement from the insult, some experience secondary injury 6–48 h later with progressive neurological manifestations.^1^ Therefore, there are still challenges in early identification of a neonate, within hours after birth resuscitation, who is likely to develop progressive HI encephalopathy and will likely benefit from hypothermia therapy.^6–8^

Conventional MRI, T_1_- and T_2_-weighted anatomical MRI and diffusion-weighted imaging (DWI), has been used to delineate the brain injury patterns in neonates at least 2 days after HI^9^^,^^10^ or to detect neuroprotection effect by hypothermia in the first days of life.^11^^,^^12^ Although certain injury patterns on MRI, such as basal ganglia and thalamic (BGT) lesion, watershed pattern of injury and bright brain,^11^^,^^13^ correlate with motor or cognitive deficits at neurodevelopmental follow-up,^9^^,^^14^ it may be difficult to define the injury during the early hours after HI. Lately, a novel MRI score was proposed, combining DWI and the most prominent injury patterns detected in the first week of life, to predict the neurodevelopmental outcome at age 2 years and school age.^15^ However, the score acquired in the first week is not useful in serving as the criteria to early identify the high-risk neonates for hypothermia treatment, which should be initiated within 6 h after birth. Sensitive neuroimaging biomarkers at early hours after HI that are related to the different brain damage severity outcomes at follow-up are needed.

Concomitant occurrence of hypoxia and ischaemia is the key pathophysiological element of brain damage after neonatal HI. Unilateral carotid artery ligation followed by systemic exposure to hypoxia, the Rice–Vannucci model, is a model widely used to investigate HI brain injury in neonatal rats and mice.^16^^,^^17^ The Rice–Vannucci model of HI has been associated with a high degree of variability with respect to the brain damage outcomes.^18–20^ Taking advantage of the variability of brain infarction outcome in this Rice–Vannucci model, we used longitudinal 7 T MRI examination to test the hypothesis that different brain damage outcomes at follow-up are related to the different neuroimaging trajectories observed early after HI. In addition, transmission electron microscopy (TEM) was applied to characterize the ultrastructural features underlying the specific areas of micro- to mesoscopic changes detected by the 7 T MRI within 6 h after HI.

## Materials and methods

This study was conducted in accordance with the recommendations of the National Institutes of Health Guidelines for Animal Research (Guide for the Care and Use of Laboratory Animals), the ARRIVE (Animal Research: Reporting *in vivo* Experiments) guidelines and the United States Public Health Service’s Policy on Humane Care and Use of Laboratory Animals. The protocol was approved by the Institutional Animal Care and Use Committee of Taipei Medical University (LAC-2016–0348 and LAC-2016–0430) and National Cheng Kung University (106109).

### HI brain injury in rat pups

On the postnatal day 10 (P 10), Sprague–Dawley male rat pups (*n* = 35) were anaesthetized with 1–2% isoflurane, followed by permanent ligation of the right common carotid artery with 5–0 surgical silk. After surgery, the pups were returned to their dams for a 1-h recovery period before 2.5 h of hypoxia. During hypoxia, the pups were placed in air-tight 500-ml containers with 37°C humidified 8% oxygen (balance, nitrogen).^21^^,^^22^ After hypoxia, the pups returned to their dams before and after MRI examination.

### Magnetic resonance imaging

Longitudinal MRI was performed in the pups (*n* = 20) by using a 7 Tesla Bruker system (PharmaScan, Bruker Corp, Ettlingen, Germany) within 6 h (*n* = 6 at 2 h, *n* = 6 at 4 h and *n* = 8 at 6 h), at 24 h and 7 days after HI. Animals were anaesthetized using 0.75% isoflurane, and the stereotaxic headpiece and holder consisting of ear and tooth bars were used to immobilize the head. Physiological conditions, including heart rate, arterial pulse extension, oxygen saturation and rectal temperature, were continually monitored and maintained within normal ranges throughout the experiment.^23–25^ A volume coil was used for RF excitation, and an array coil was used for receiving signal. Initial localization scans were performed, and T_2_-weighted images using a Rapid Acquisition with Relaxation Enhancement (RARE) sequence with TR/TE = 3000/45 ms, FOV = 2.0 × 2.0 cm, matrix size = 320 × 320, 16 slices and slice thickness of 0.75 mm, was performed to acquire anatomical images for rodent models.^23^^,^^25^ Diffusion data were acquired with the same geometry by using the four-shot spin-echo EPI with TR/TE = 4000/38 ms, matrix size = 96 × 96, *δ*/Δ  =  2.3/22 ms, number of b0 = 5, number of directions = 30, b-value = 1000 s/mm^2^ and number of averages = 2.

Image analysis including skull stripping and motion correction or coregistration across time points and subjects was performed using Statistical Parametric Mapping and a custom Matlab (MathWorks Inc., Natick, MA) script published previously.^23^^,^^24^^,^^26^ The severity of brain damage outcome determined using the MRI was based the MR-derived hemispheric volume loss defined as the brain volume in the ipsilateral cerebral hemisphere divided by the contralateral cerebral hemisphere in T_2_-weighted images acquired at 7 days after HI (P17). The oedema volume in the ipsilateral hemisphere was excluded to adequately compare with the histological data. Apparent diffusion coefficient (ADC) maps were calculated from the diffusion tensor images. The threshold of 70% of the contralateral homologous brain was used to extract the ADC deficit area. The threshold was selected based on the values in studied of stroke in rodents.^23^^,^^27^ At 7 days after HI, the deficit area was delineated as stated earlier for the animals without oedema transformation; for the animals with oedema, the deficit area was further calculated based on hyperintense signals (≥2 SD of contralateral normal cortical tissue) in T_2_-weighted images.^28^ The averaged ADC values within the deficit volumes were calculated. The ratio of ADC deficit was defined as the ADC deficit volume divided by the volume of the ipsilateral cerebral hemisphere. Given the potential ventriculomegaly long after HI, the volume of ventricles was masked out in ADC maps 7 days after HI (Supplementary Fig. 1A) when calculating the parenchymal volume. The hemispheric volume change was defined as the volume of the ipsilateral parenchymal volume divided by that of the contralateral parenchymal volume. Regions of interests (ROIs) were placed in the parietal cortex, thalamus, hippocampus, striatum and corpus callosum in the ipsilateral hemisphere (Supplementary Fig. 1B–D) in the fractional anisotropy map^29^ to reveal the temporal trajectory of regional changes after HI among the pups that became the three brain damage outcome groups at follow-up. All threshold-deﬁned results were visually veriﬁed by two experienced investigators blinded to the experimental groups.

### Brain damage outcome measurement by histopathology

Overall, 24 male rat pups from 6 dams (20 rat pups with longitudinal MRI and another 4 pups for histopathology only) were used for HI outcome measurement by using histopathology on P17 to establish the different severities of brain damage outcomes in the Rice–Vannucci rat pup model of HI. The brain damage severity outcome was determined using the ipsilateral cerebral hemispheric volume loss compared with the contralateral cerebral hemisphere volume. After being anaesthetized with isoflurane, the rats were perfused transcardially with saline and then cold phosphate-buffered 4% paraformaldehyde. The brain sections (10-µm thick) that stained with cresyl violet staining were scanned by Aperio slides scanner (ScanScope CS; Leica Biosystem) for calculating the brain damage areas. Bilateral cerebral hemispheres were manually assessed by tracing the histological area by using a computerized image analysis system (ImagePro Plus 6.0, Media Cybernetics) linked to a Nikon E400 fluorescence microscope (Tokyo, Japan). The total cross-sectional area in each brain region was calculated at 12 predetermined coronal sections. The cerebral volume loss in the ipsilateral versus the contralateral hemisphere was calculated as follows: (contralateral volume––ipsilateral volume)/(contralateral volume).^30^^,^^31^ The ipsilateral cerebral hemispheric volume loss compared with the contralateral cerebral hemisphere with Nissl staining was used to represent the degree of brain damage severity after HI. Based on the degree of the ipsilateral cerebral hemispheric volume loss, the mild damage outcome group was defined as the group with ipsilateral cerebral hemisphere volume loss of less than 15%, moderate damage outcome group as the group with a hemispheric volume loss between 15% and 45% and severe damage outcome group as the group with a hemispheric volume loss greater than 45%.

### Transmission electron microscopy

Different ultrastructural changes after HI were examined in a subset of 13 male pups (*n* = 11 after HI, and *n* = 2 sham control pups without CCA occlusion) by using the TEM. After MRI scan within 6 h after HI, the brain section was obtained from the parietal cortex and corpus callosum at 3.8 mm posterior to the bregma in the ipsilateral cerebral hemisphere and trimmed into 1–2 mm slices by using the acrylic brain matrices. The samples were post-fixed overnight in 2% paraformaldehyde and 2.5% glutaraldehyde in 0.2 M cacodylate. All sections were then rinsed in phosphate buffer, post-fixed with 1% osmium tetroxide (OsO_4_) dissolved in 0.1 M phosphate buffer, dehydrated with alcohol and embedded in Epon-Araldite mixture. Ultra-thin sections were obtained using a diamond knife. Tissue sections without routine double-staining were examined and photographed using the Hitachi 7700 electron microscope attached with a CCD camera.^32^ The ultrastructural cytologic characteristics, including the neurons in the cortex and the myelin sheath and oligodendrocytes in the white matter, were assessed.

### Statistical analysis

Statistical analysis was performed using SPSS software (IBM SPSS statistics 21, IBM Corp, Armonk, NY). Differences in the deficit volumes, ADC values, ADC deficit ratio, hemispheric volume and regional ADC values among the three different brain damage outcome groups were compared at multiple time points using unpaired 2-way ANOVA, followed by Tukey’s honestly significant difference and Scheffe *post hoc* tests that were performed to analyse the data passing and not passing the homogeneity test, respectively. A Spearman’s correlation was performed to examine the monotonic relationship between the IHC-derived and MR-derived hemispheric volume loss. A Pearson’s correlation test was used to detect significant correlations between ADC changes in the acute phase and the final hemispheric volume loss. The significance level was set at *P *<* *0.05. Error bars were STD.

### Data availability

Raw image data were acquired at Taipei Medical University. Derived data supporting the finding of the current study will be shared by request from a qualified academic investigator for the purpose of replicating experimental and analytical procedures, as well as the results presented in the article.

## Results

### The spectrum of brain damage severity outcomes after HI determined using histopathology and MRI

**Figure 1:**
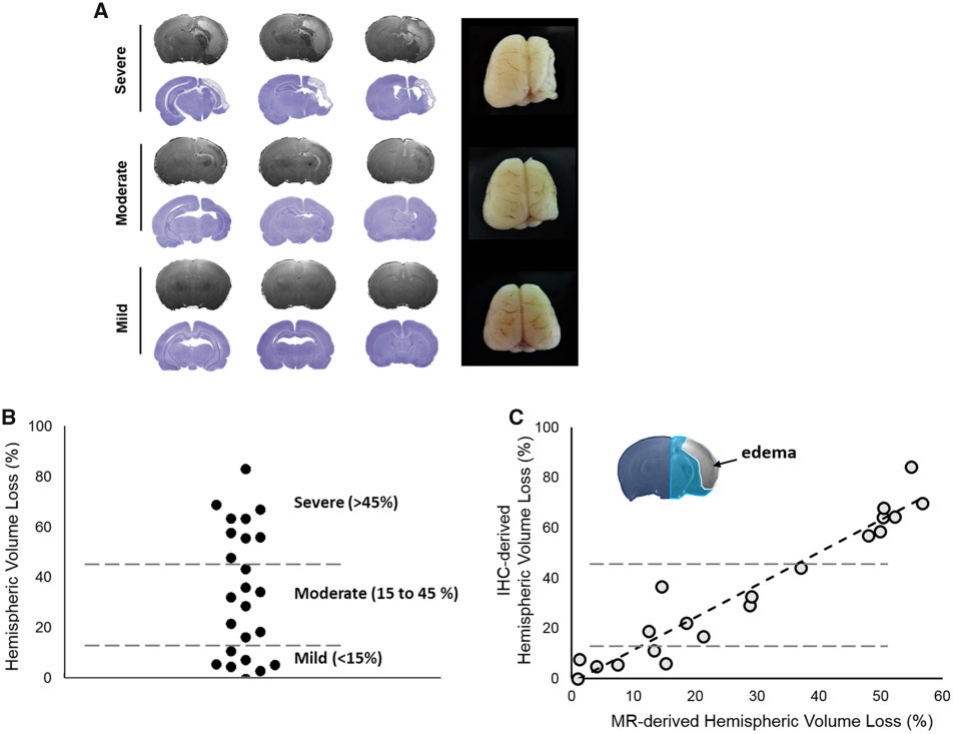
**The spectrum of different brain damage severity outcomes after the same duration of HI determined using histopathology and MRI.** (**A**) Different degrees of brain damage outcomes, namely mild, moderate and severe, were observed using the T_2_-weighted images (upper panel), Nissl-stained histopathology (lower panel) and gross anatomy (right panel) 7 days after the same duration of HI. (**B**) The ipsilateral cerebral hemispheric volume loss compared with the contralateral cerebral hemisphere by using histopathology was used to represent the spectrum of brain damage severity outcomes after HI. *N* = 24 for histopathology. (**C**) A Raster plot of brain damage severity outcome in the relationship between the percentages of hemispheric volume reduction measured by MRI and that by histopathology (IHC). The linear regression line was plotted as the overall regression was significant (*P *<* *0.001) determined by using a Spearman’s correlation test. The inset shows the exclusion of the oedema formation region during the calculation of MR-derived brain damage outcomes based on T_2_-weighted images. Light blue, ipsilateral hemisphere; dark blue, contralateral hemisphere. *N* = 20 for MRI.

First, histopathology was used to determine the spectrum of brain damage severity outcomes 7 days after the same duration of HI in the male rat pups (Fig. 1A). Based on the percentage of ipsilateral cerebral hemispheric volume loss, there were 29.2% (7/24) of the pups in the mild damage outcome group (volume loss less than 15%), 33.3% (8/24) in the moderate damage outcome group (volume loss between 15% and 45%) and 37.5% (9/24) in the severe damage outcome group (volume loss greater than 45%) (Fig. 1B).

The correlative MRI showed significant volume loss in the ipsilateral cerebral hemisphere was observed in the moderate and severe outcome groups compared with the somewhat symmetrical cerebral hemispheres without prominent lesion in the mild outcome group (Fig. 1A). T_2_-weighted MR images also demarcated the area of oedema formation in the ipsilateral hemisphere of the severe outcome group (Fig. 1A). The percentages of volume reduction in the ipsilateral cerebral hemisphere 7 days after HI determined using the T_2_-weighted images were significantly correlated with those measured using conventional Nissl-stained histopathology (*r *=* *0.943, *P *<* *0.001), indicating the feasibility of T_2_-weighted MR images in measuring the brain damage outcome after HI (Fig. 1C).

### Longitudinal MRI assessment after HI

**Figure 2:**
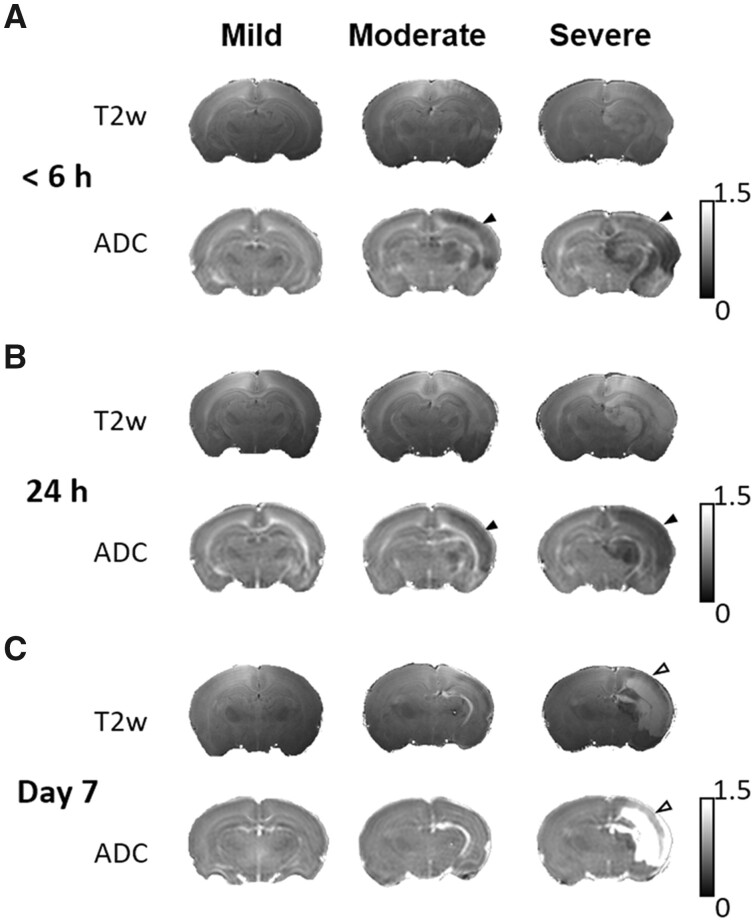
**Longitudinal changes in diffusion and T_2_-weighted MR images at 6 h, 24 h and 7 days after HI in different brain damage severity outcome groups.** Representative ADC map and T_2_-weighted images within 6 h in **A**, at 24 h in **B** and 7 days in **C** after HI in the mild, moderate and severe outcome groups. Although no ADC change was observed in the mild outcome group, the ADC deficit region (black arrowheads) observed in the ipsilateral cerebral cortex and the subcortical area within 6 h after HI, and expanded at 24 h after HI in the moderate and severe outcome groups. A significant reduction of the ipsilateral hemispheric volume was observed at 7 days after HI in the moderate and severe outcome groups. Oedema formation (white arrowheads) was evident in both T_2_-weighted images and the ADC map at 7 days in the severe outcome group.

Longitudinal changes in diffusion and T_2_-weighted MR images after HI leading to the mild, moderate and severe damage outcome groups were observed, respectively (Fig. 2). Even though no change was observed in the mild outcome group within 6 h after HI, the hypointense signals of ADC deficit regions were seen in the ipsilateral cortex, subcortical hippocampus and striatum areas in the moderate and severe outcome groups. Hyperintensity in T_2_-weighted images was sparsely revealed in some animals (5/9) in the severe outcome group (Fig. 2A). However, no significant difference was observed in signal intensity compared with the contralateral hemisphere. At 24 h after HI, the regions with decreased ADC signals expanded and became more prominent in the moderate and severe outcome groups. Expanded regions with hyperintensity in T_2_-weighted images were also observed in the severe outcome group (Fig. 2B). At 7 days after HI, oedema transformation with hyperintensity in both T_2_-weighted images and ADC map developed in the severe outcome group (Fig. 2C). A smaller ipsilateral cerebral hemisphere was observed in both moderate and severe outcome groups.

**Figure 3:**
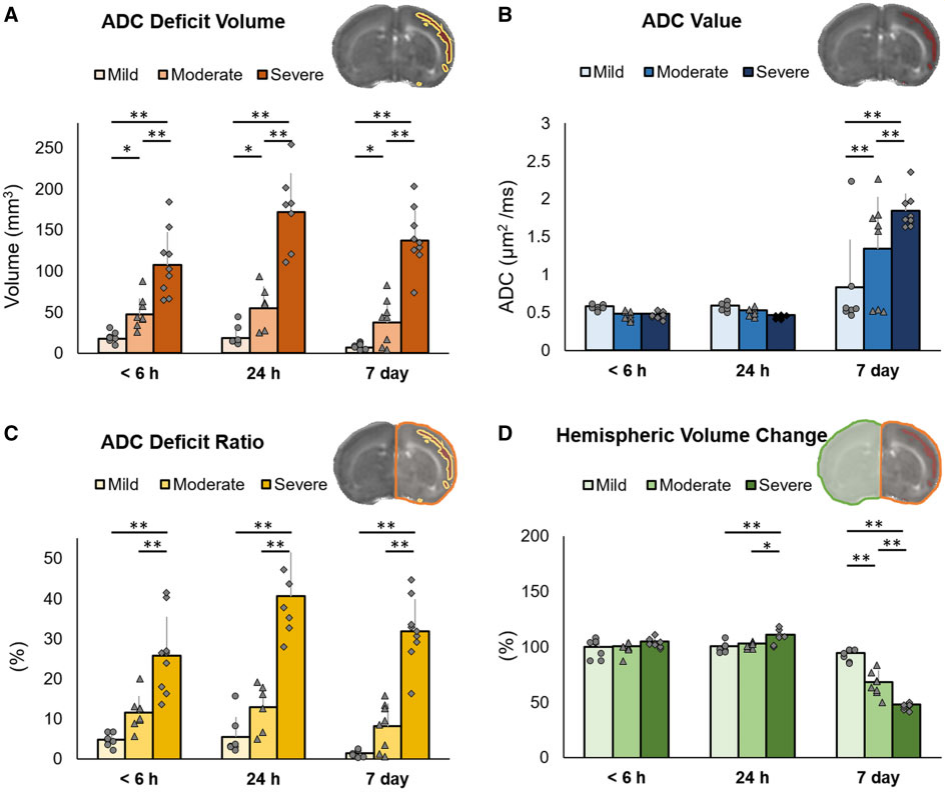
**The difference of changes in quantitative MRI characteristics after HI among the mild (*N* = 7), moderate (*N* = 8) and severe (*N* = 9) damage outcome groups at each time point after HI.** ADC-derived deficit volume in **A**, mean ADC values in **B**, ADC-derived deficit ratio in **C** and **D** hemispheric volume changes defined using T_2_-weighted images within 6 h, at 24 h and 7 days after HI in **D**. The inset images labelled the ADC deficit volume (yellow contour); ADC deficit region (red); ADC-derived deficit ratio: ADC deficit volume (yellow contour)/ipsilateral hemispheric volume (orange contour); hemispheric volume change: The volume in the ipsilateral (orange contour)/contralateral hemispheres (green contour), respectively. The error bars were standard deviation. Significance level (*P <* *0.05 and **P <* *0.005) among groups at each time point was determined by using an unpaired 2-way ANOVA with *post hoc* tests (ADC deficit volume *F*_4,66_ = 2.884, *P *=* *0.03; ADC values *F*_4,66_ = 7.697, *P *<* *0.001; ADC deficit ratio *F*_4,66_ = 2.799, *P *=* *0.03 and hemispheric volume change *F*_4,66_ = 48.08, *P *<* *0.001).

Two-way ANOVA revealed that quantitative MRI characteristics (ADC deficit volume *F*_4,66_ = 2.884, *P *=* *0.03; ADC values *F*_4,66_ = 7.697, *P *<* *0.001; ADC deficit ratio *F*_4,66_ = 2.799, *P *=* *0.03 and hemispheric volume change *F*_4,66_ = 48.08, *P *<* *0.001) was significantly affected by HI outcomes and the time points after HI. Significant differences were observed among the three groups regarding the increases in the ADC deficit volume (Fig. 3A) and deficit ratios (Fig. 3C) within 6 h, at 24 h and 7 days after HI, with the highest increases observed in the severe outcome group. Notably, significant differences were already observed regarding the ADC deficit volume between the mild and severe outcome groups, between the mild and moderate outcome groups and between the moderate and severe outcome groups within 6 h after HI. By contrast, the increase in ADC values (Fig. 3B) was not significant until 7 days after HI in the three groups. Significant changes were observed among the three groups regarding the ipsilateral cerebral hemispheric volume, which increased at 24 h in the severe outcome group and decreased at 7 days after HI in the moderate and severe outcome groups (Fig. 3D). In addition to significant differences in MRI characteristics among groups, we observed longitudinal changes in these characteristics at different time points in each group. Although significant increases in the ADC deficit volume and deficit ratios were observed in the severe outcome group at 24 h (Supplementary Fig. 2A and C), the deficit volume decreased at 7 days. The significantly higher ADC values but with a significantly decreased hemispheric volume were noted in both the moderate and severe outcome groups at 7 days after HI (Supplementary Fig. 2B and D).

**Figure 4:**
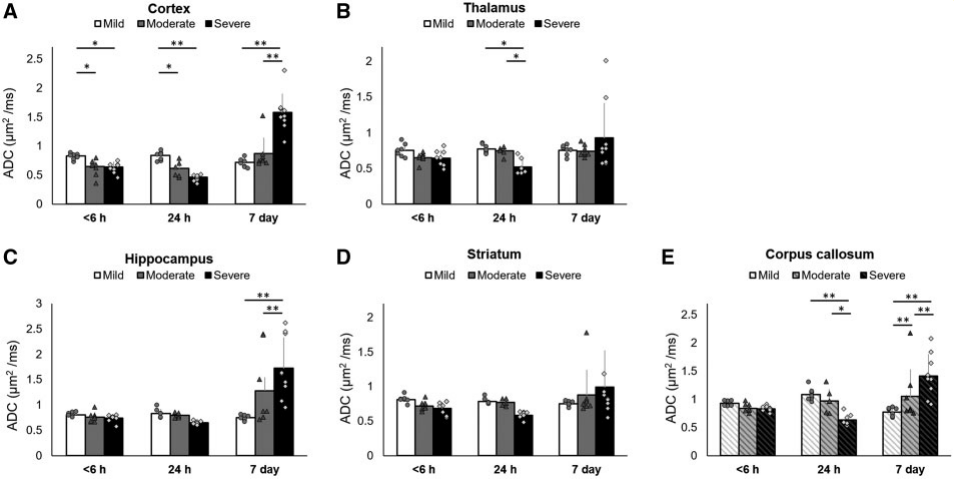
**ROIs analysis of the regional ADC values in different brain areas among the mild (*N* = 7), moderate (*N* = 8) and severe (*N* = 8) damage outcome groups at each time point after HI.** The mean ADC values in the ipsilateral cortex in **A**, thalamus in **B**, hippocampus in **C**, striatum in **D** and corpus callosum in **E** within 6 h, at 24 h and 7 days after HI. The error bars were standard deviation. Significance level (*P <* *0.05 and **P <* *0.005) among groups at each time point was determined by using an unpaired 2-way ANOVA with *post hoc* tests (the cortex *F*_4,66_ = 29.592, *P *<* *0.001; the thalamus *F*_4,66_ = 2.881, *P = *0.03; the hippocampus *F*_4,66_ = 6.673, *P *<* *0.001; the striatum *F*_4,66_ = 1.926, *P *=* *0.118 and the corpus callosum *F*_4,66_ = 10.590, *P *<* *0.001).

To further elucidate the temporal changes in regional ADC values among three outcome groups, we performed ROI analysis in different brain areas (the cortex *F*_4,66_ = 29.592, *P *<* *0.001; the thalamus *F*_4,66_ = 2.881, *P *=* *0.03; the hippocampus *F*_4,66_ = 6.673, *P *<* *0.001; the striatum *F*_4,66_ = 1.926, *P *=* *0.118 and the corpus callosum *F*_4,66_ = 10.590, *P *<* *0.001). Within 6 h after HI, the only significantly lower ADC was observed in the cortex of the pups that later became the moderate and severe outcome groups compared with that of the pups that became the mild outcome group (Fig. 4A). At 24 h after HI, in addition to the cortex (Fig. 4A), significantly lower ADC was also involved in the thalamus and corpus callosum (Fig. 4B and E) in the severe outcome group, suggesting later alteration in ADC in these two regions in the severely affected group. At 7 days after HI, significantly higher ADC was observed in the cortex, hippocampus and corpus callosum (Fig. 4A, C and E), especially in the severe outcome group. No significant ADC trajectory changes were observed in the striatum after HI among the three outcome groups (Fig. 4D).

### ADC-derived parameters early after HI correlated well with the brain damage severity outcomes

**Figure 5:**
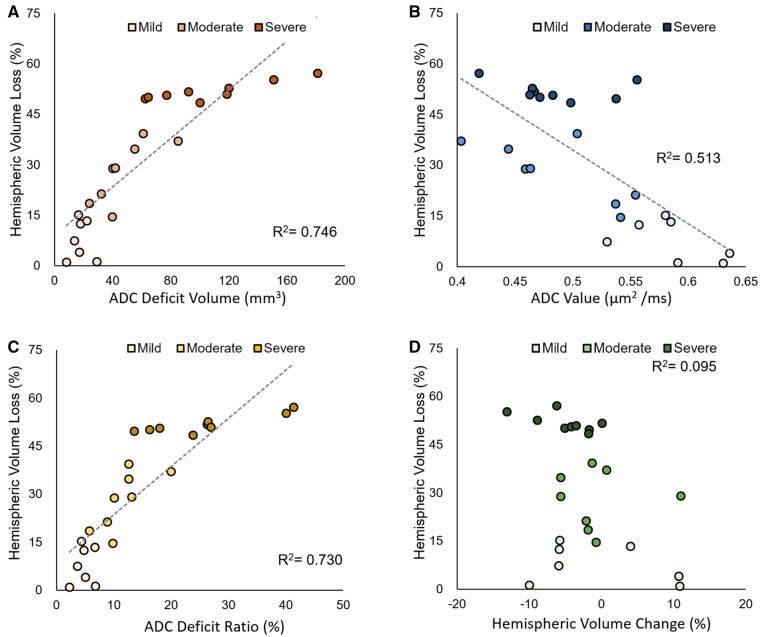
**Correlation between the MRI characteristics within 6 h after HI and the brain damage severity outcome 7 days after HI.** The respective raster plot on the relationship between the ADC-derived deficit volume in **A**, ADC values in **B**, ADC-derived deficit ratio in **C** and cerebral hemispheric volume changes defined using T_2_-weighted images in **D** within 6 h after HI versus the cerebral volume loss 7 days after HI. The severity of brain damage was categorized by the percentages of ipsilateral cerebral hemispheric volume loss measured using T_2_-weighted images at 7 days after HI. The linear regression line was plotted (ADC-derived deficit volume, ADC values, ADC deficit ratios) if the overall regression was significant (*P *<* *0.05) determined by using a Pearson’s correlation test.

We then correlated the early quantitative MRI characteristics (ADC deficit volume, ADC values, ADC deficit ratio and hemispheric volume change) in the ipsilateral cerebral hemisphere noted within 6 h after HI with the brain damage severity outcome defined using T_2_-weighted images at 7 days after HI. We determined that the ADC deficit volume (Fig. 5A), ADC values (Fig. 5B) and ADC deficit ratios (Fig. 5C) were highly correlated with the brain damage severity outcome measured using the percentage of ipsilateral cerebral hemispheric volume loss at 7 days after HI. However, the acute hemispheric volume changes (the ipsilateral hemisphere divided by the contralateral hemisphere) showed no correlation (Fig. 5D). A larger ADC deficit volume (regions displaying <70% of ADC signals) and ADC deficit ratios and lower ADC values were associated with more severe brain damage outcomes.

### Ultrastructural correlates in the cortex and white matter underlying the areas of early ADC changes after HI

**Figure 6:**
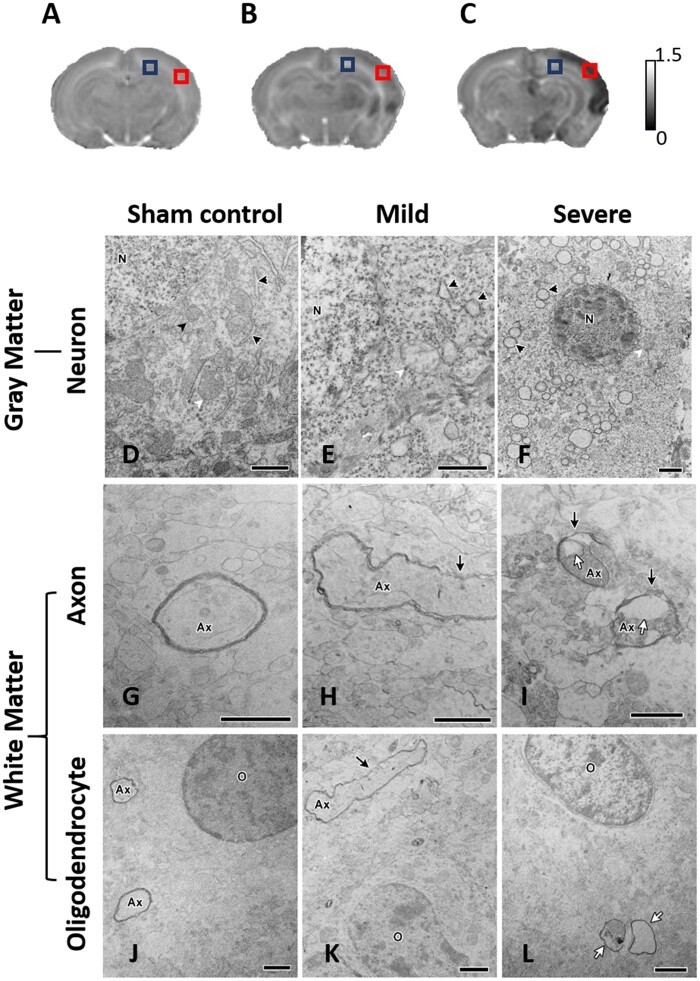
**The ultrastructural characteristics in the cortex (neurons) and white matter (axons and oligodendrocytes) underlying the ADC changes noted early after HI that correlated with different brain damage severity outcomes.** The ADC map in the sham control in **A** and within 6 h after HI that correlated with mild damage in **B** and severe damage in **C** outcome groups. The red and blue boxes indicated the sample of TEM in the cortex and white matter, respectively. Transmission electron microscopy showed the neurons in **D–F** in the cortex, and the myelinated axons in **G–I** and oligodendrocytes in **J–L** in the white matter in the sham control, mild and severe outcome groups. Compared with the cortical neurons in the sham control group in **D**, the neuron showed dilation of rER (black arrowheads) and swollen mitochondria (white arrowheads) in the mild outcome group in **E**, but massive swelling of rER (black arrowheads) and the degenerated mitochondria (white arrowheads) accompanied with degradation of nucleus in the severe outcome group in **F**. Compared with the white matter in the sham control group in **G**, demyelination (black arrows) was noted in the mild in **H** and severe in **I** outcome group, whereas prominent axon detraction (white arrows) was only observed in the severe outcome group in **I**. By contrast, the morphology of oligodendrocyte was similar among the sham control, mild and severe outcome groups in **J, K** and **I**. N, the nucleus of the neuron; Axe, axon; O, oligodendrocyte. The scale bar, 1 µm.

We then used TEM to characterize the ultrastructural characteristics in the cerebral cortex and white matter underlying the areas of ADC changes detected within 6 h after HI that predicted to correlate with the different severity outcome groups. The animals were classified into different severity outcome groups based on their ADC-derived parameters (Supplementary Fig. 3). Compared with the sham control group (Fig. 6A and D), the apoptotic-like degenerating neurons characterized by fragmented nuclei, swollen rough endoplasmic reticulum (rER) and degenerating mitochondria were observed in the cortex area showing significant ADC deficit changes early after HI that were highly associated with severe damage outcome (Fig. 6C and F). By contrast, the cortex with a small ADC deficit region that was associated with the mild damage outcome revealed rER dilation and swollen mitochondria (Fig. 6B and E). Prominent myelin loss and axon detraction were observed in the white matter areas showing marked ADC deficits that were associated with severe damage outcomes (Fig. 6C and I). On the other hand, the white matter region with mild ADC deficit that correlated with mild damage outcome only revealed myelin detachment (Fig. 6B and H). Although significant demyelination was observed early after HI, the morphology of oligodendrocytes was preserved among the three outcome groups (Fig. 6J–L).

## Discussion

The current study demonstrated the spectrum of different brain damage severity outcomes by using the well-established Rice–Vannucci model of neonatal HI. Using longitudinal 7 T MRI examination on this model with diverse brain damage outcomes, we characterized the different neuroimaging trajectory patterns early after HI leading to different brain damage outcomes. The distinct patterns of cerebral injury were recognized as early as within 6 h after HI based on the ADC deficit volume and deficit ratio. These patterns progressed at 24 h and 7 days after HI and evolved into the three outcome groups. Notably, the early ADC characteristics (ADC deficit volume, ADC values and ADC deficit ratios) observed within 6 h after HI were highly correlated with the brain damage severity outcomes 7 days after HI. In addition, the early changes in ADC signals associated with different brain damage outcomes at follow-up were correlated well with the distinct ultrastructural organelle changes showing restricted cellular water diffusion in the intra- and extra-cellular spaces in the cerebral cortex and white matter. Overall, our findings suggest that the distinctive ADC patterns and parameters detected early after HI that highly associated with different brain injury progression and damage severity outcomes may be useful to early identify high-risk neonate for hypothermia therapy after HI.

To imitate HI brain damage due to failure of cerebral perfusion to the foetus caused by uterine, placental, or umbilical cord compromise prior to or during delivery,^33^ different animal models have been developed.^16^^,^^17^^,^^34^^,^^35^ In rat pups, unilateral carotid artery ligation combined with systemic hypoxia is associated with decreased cerebral blood flow and ischaemic brain damage in the ipsilateral hemisphere,^17^ making the Rice–Vannucci model the most commonly used rodent model for neonatal HI encephalopathy. The pathological changes induced by this model involving the striatum, hippocampus and cortical areas are quite similar to that in human neonates with moderate and severe HI encephalopathy. However, a high degree of variability in the infarct volume is one limitation of this model.^18^ One factor affecting the outcome variability in this model maybe related to the interval between ligating the common carotid artery and exposure to systemic hypoxia.^21^^,^^36^ Another factor may involve the occurrence of communicational blood flow within the circle of Willis and the branches of external carotid arteries following common carotid artery occlusion since the occlusion of the external and common carotid arteries greatly reduces infarct volume variability by limiting the collateral blood flow.^18^ Moreover, our previous studies observed a broad spectrum of brain damage severity outcomes after the same duration of HI in this model.^22^^,^^37^ Although significant oedema changes along with volume reduction were observed in the severe outcome group, nearly no hemispheric volume change was shown in the mild outcome group. We took advantage of this model of diverse damage outcomes that mimics the mild, moderate and severe HI encephalopathy in new-borns to show that different brain damage outcomes were related to different neuroimaging trajectories observed early after HI.

Clinically, predictive MR markers of adverse outcomes after neonatal HI have been proposed. A meta-analysis by Thayyil et al.^10^ revealed that the lactate to N-acetyl aspartate (LAC/NAA) ratio detected using proton MR spectroscopy in the deep grey matter at days 1–30 after birth surpassed conventional MRI with the highest specificity (0.95) and sensitivity (0.82) in predicting unfavourable outcomes at age 12 months. Lately, Shankaran et al.^38^ determined that the MRI scores based on the brain injury pattern detected at an average of 15 days after birth were associated with the outcome of death or IQ <70 at the age of 6–7 years. However, in this hypothermia era, it is impossible to translate these MR biomarkers to identify high-risk new-borns within 6 h after birth for providing them with hypothermia therapy. Experimentally, while Doman et al.,^12^ showed that the T_2_-weighted imaging-based MRI scores obtained 3 h after HI could reliably assess initial brain injury and predict brain damage outcome after hypothermia therapy in mice, the early change in diffusion characteristics after HI has not been well reported yet. In our study, the earliest MRI was performed at 2–6 h to monitor the evolution of multi-parametric MR signal after HI with subsequent scans at 24 h and 7 days after HI. Using the neonatal HI model with different brain damage severity outcomes under the same duration of HI, we demonstrated that the quantitative MR features not only reflect the various brain damage outcomes but also reveal a significant correlation between the ADC characteristics within 6 h after HI and brain damage outcomes at follow-up.

Our study showed that within 6 h after HI, the severe outcome group had a significantly larger ADC-derived deficit volume and ADC deficit ratio compared with the mild or moderate outcome groups. Moreover, the moderate outcome group had a significantly larger ADC deficit volume than the mild outcome group. Specifically, a striking discrepancy was already observed in the brain injury areas spreading across the cerebral cortex, hippocampus, thalamus, striatum, amygdala, corpus callosum and internal capsule at the same time point early after HI among the three outcome groups. Significant correlations were observed between the early ADC deficit volume and ADC deficit ratio within 6 h after HI and the brain damage severity outcome measured at 7 days after HI. Of note, considerable variety of ADC deficit volume was prominent after 24 h and 7 days after HI, which is concordant with the findings of clinical studies revealing different brain injury patterns observed using DWI or diffusion tensor imaging 2 days after HI.^13^^,^^15^^,^^38^

We observed the different longitudinal changes in cerebral injury depicted by ADC and hemispheric volume changes at 6 h, 24 h and 7 days after HI in the three different outcome groups. In the moderate and severe outcome groups, significant increases in ADC values were accompanied by a significant loss of ipsilateral hemisphere tissue 7 days after HI. In the severe outcome group, a significantly larger hemispheric volume change detected at 24 h after HI corresponded to a significantly smaller hemispheric volume 7 days after HI. Furthermore, previous studies of neonatal HI have revealed large ADC values and significant cerebral tissue loss weeks after the insult, primarily owing to the formation of porencephalic cysts.^39–41^ Our results provide evidence indicating that more severe vasogenic progress after HI may lead to subsequent cerebral oedema transformation. The change in ADC values because of vasogenic oedema after HI may surpass the decreases in ADC caused by the myelin maturation and concentration in the developing brain.^42^

The underlying ultrastructural changes in the cortex and white matter were used to characterize the early ADC changes within 6 h after HI that correlated with different brain damage severity outcomes at follow-up. We observed that the areas with significant ADC deficits noted early after HI already had marked apoptotic-like degenerating neurons characterized by fragmented nuclei, swollen rER and degenerating mitochondria in the cortex and prominent myelin loss and axon detraction in the white matter. By contrast, the area of early ADC deficits observed in the mild outcome group showed only rER dilation and swollen mitochondria in the cortical neurons and detachment of myelin in the white matter. The results are consistent with the findings from focal ischaemic stroke showing large numbers of pale neurons with cytoplasmic vacuoles which correlated with lower ADC values in the regions of the secondary neural degeneration.^43^ In contrast to the vulnerable white matter after ischaemic stroke in adult rats,^44^ our results are in line with other studies showing no change in myelinated axons in the corpus callosum underneath the sensorimotor cortex^45^ early after neonatal HI. More importantly, in the rat pup model of HI, the mechanism of white matter injury involves maturation-dependent selective vulnerability in the oligodendrocyte lineage.^46–48^ The timing of appearance of late oligodendrocyte progenitors is the major developmental factor accounts for the susceptibility of neonatal white matter injury. Late oligodendrocyte progenitors are the major oligodendrocyte lineage stage killed by apoptosis, whereas early oligodendrocyte progenitors and more mature oligodendrocytes are highly resistant. Although the morphology and numbers of oligodendrocytes were not altered early post-HI, our results showed demyelination in the mild outcome group and detraction of axons in both mild and severe outcome groups, suggesting injury in the cellular process of oligodendrocytes might have begun early after HI.

**Figure 7:**
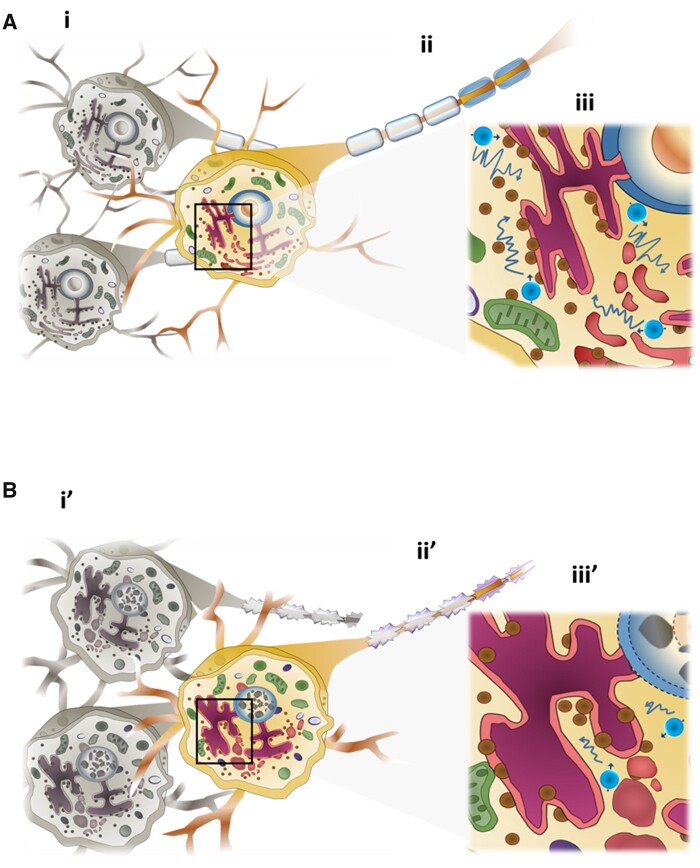
**Hypothesis schema showing restricted cellular water diffusion underlying the areas of low ADC values in the cortex and white matter within 6 h after HI.** (**A**) In the intact brain, water molecules are freely diffused in the extracellular (**i**) and intracellular space (**iii**), as well as along the myelinated axons in the white matter (**ii**). **(B)** Early after HI, cell swelling with decreased extracellular space volume interferes with the water diffusion (**i’**). In the myelinated axons, the degeneration of myelin and the detraction of axons obstruct diffusion of myelin water (**ii’**). In addition, aggregation of chromatin in the nucleus and massive swelling of organelles in the cytoplasm may hinder the intracellular free water diffusion (**iii’**).

Normal water diffusion in the brain is modulated by free water diffusion in the extra- and intra-cellular spaces as well as by water movement along the myelinated axons in the white matter, which are all affected by the cell size, cell density and orientation.^49^ The underlying causes of restricted water diffusion observed in the ADC deficit regions within 6 h after HI may include cell swelling that increases the tortuosity in the extracellular space,^50^^,^^51^ aggregation of chromatin in the nucleus and massive swelling of organelles in the cytoplasm that hinder the free water diffusion in the intracellular space^43^ and demyelination and axon detraction that obstruct diffusion of myelin water (Fig. 7).^49^ Notably, the mesoscopic structural changes detected early after HI using 7 T MRI in this study that correlated with various ultrastructural characteristics of restricted cellular water diffusion in the intra- and extra-cellular spaces of the cerebral cortex and white matter could have already determined the brain damage severity outcome at follow-up. Nevertheless, even though the neurite beading—a recently proposed pathological mechanism that causes decreased ADC after ischaemic stroke^52^—has not been observed after HI, future studies combining the analysis of tensor metrics and microscopy that focus on the neurite may clearly illustrate the changes in cell-membrane morphology and the subsequent alteration of water mobility.

This study had some limitations. First, as no gold standard of the ADC threshold exists for probing the early HI lesion in neonatal pups, the ADC deficit area presented herein was based on the proposed threshold in adult rodents after ischaemic stroke.^23^^,^^24^ We acknowledge that using the 70% threshold of the ADC values in the contralateral hemisphere might extract the ADC deficit area biased to the relative severe lesions. Future work using voxel-by-voxel analysis may better illustrate the subtle changes in specific regions after HI. In addition, the quantification of ADC changes could be affected by potential partial volume effects, especially in pups with mild damage outcomes with relatively small deficit regions. Our study demonstrated that early changes in diffusion MR characteristics correlated with different brain damage outcomes after HI, but we only studied the most widely used diffusion index in clinical practice, namely ADC-related parameters. Therefore, further studies related to the whole spectrum of diffusion metrics^41^^,^^53^ or using multi-shell diffusion MRI^54^^,^^55^ may better illustrate the complicated microstructures or crossing fibres, which may facilitate the translation of the early neuroimage biomarkers to human trials.

## Conclusion

In conclusion, this study found that different ADC patterns and parameters depicted in the early period after neonatal HI are highly associated with different brain injury progression trajectories and brain damage severity outcomes. These early neuroimaging findings may contribute to the identification of high-risk new-borns who are likely to develop progressive HI encephalopathy within hours after birth for hypothermia therapy.

## Supplementary material

Supplementary material is available at *Brain Communications* online.

## Supplementary Material

fcab048_Supplementary_Material

## Abbreviations

ADC =: apparent diffusion coefficient
DWI =: diffusion-weighted imaging
HI =: hypoxic ischaemia
rER =: rough endoplasmic reticulum
ROI =: regions of interests
TEM =: transmission electric spectroscopy
