# Supplementary material for: Early neuroimaging and ultrastructural correlates of injury outcome after neonatal hypoxic-ischaemia
Source: Brain Commun. 2021 Mar 26;3(2):fcab048. doi: 10.1093/braincomms/fcab048 (PMC8103732; doi:10.1093/braincomms/fcab048)
Supplement: fcab048_Supplementary_Material [file fcab048_supplementary_material.docx]

**Supplementary Material**


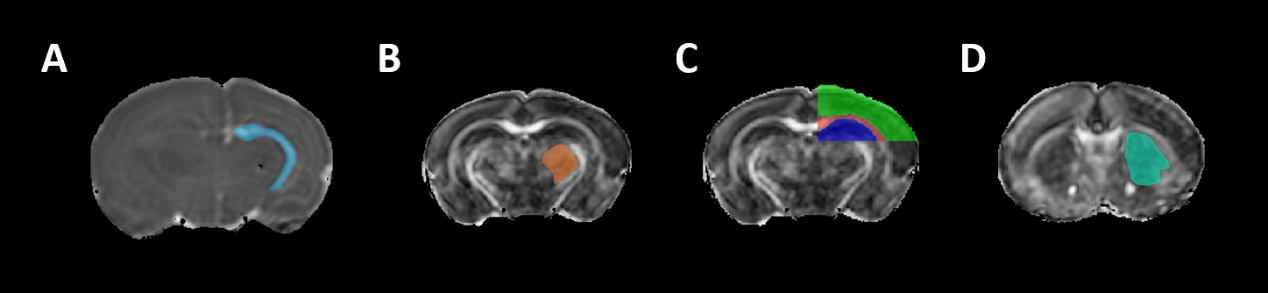


**Supplementary Figure 1** **Representative regions of interest (ROIs) in the animal of the moderate outcome group.** (**A**) The enlarged ventricle in the ipsilateral hemisphere at 7 days after HI were labeled and subtracted for calculation of the final hemispheric volume loss. The ROIs in the thalamus (**B**), cortex (green), corpus callosum (red), hippocampus (blue) (**C**), and striatum (**D**) were labeled in the FA map. The mean ADC values in the ROIs were extracted to show the regional and temporal change after HI.


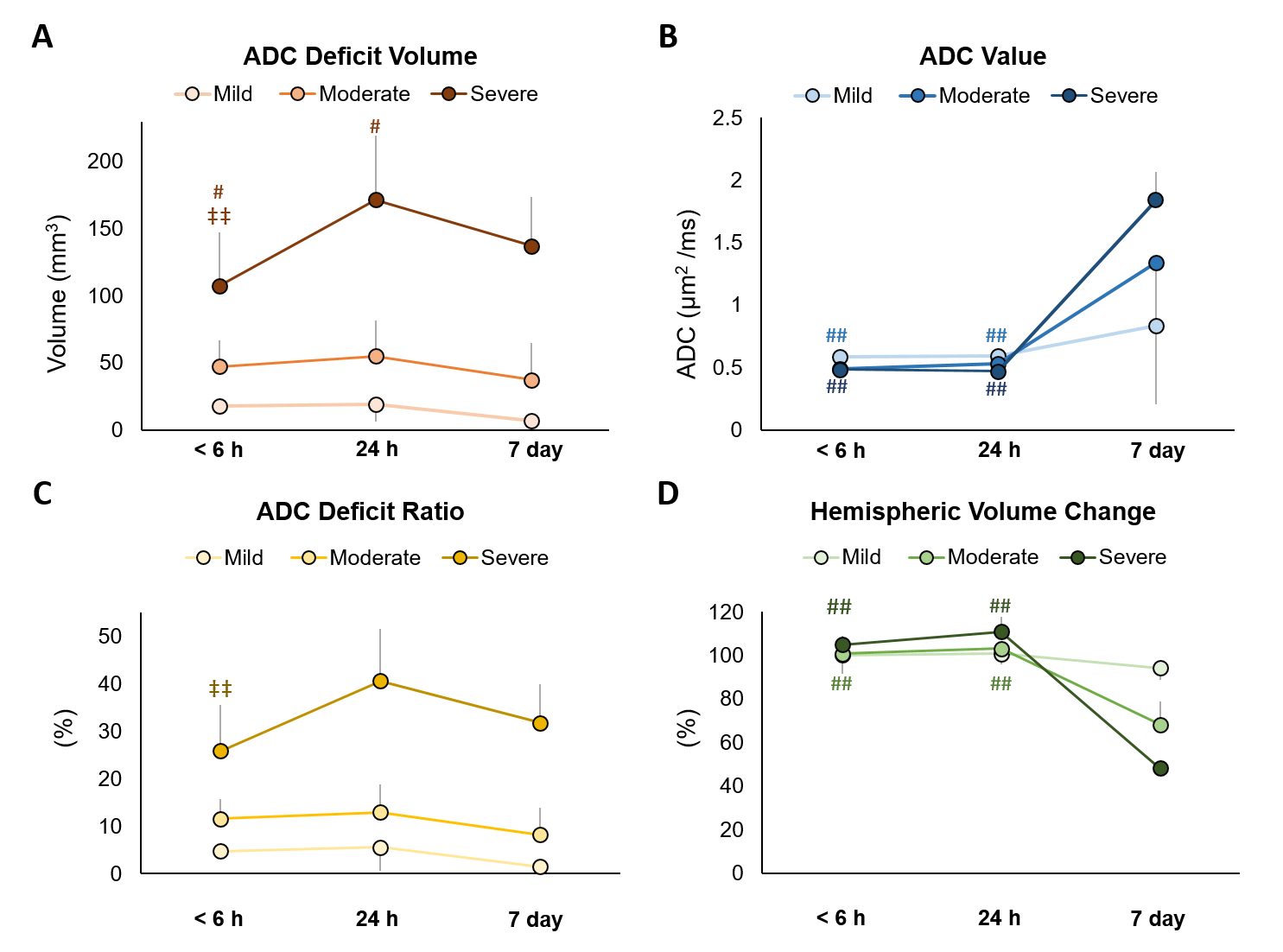


**Supplementary Figure 2 Longitudinal changes in the quantitative MRI characteristics after HI in the mild, moderate, and severe damage outcome groups**. (**A**) ADC derived deficit volume, (**B**) mean ADC values, (**C**) ADC derived deficit ratio, and (**D**) hemispheric volume changes defined using T_2_-weighted images within 6 hours, at 24 hours, and 7 days after HI. ‡ (*P* < 0.05) and ‡‡ (*P* < 0.005) indicated significance from 24 hours after HI. # (*P* < 0.05) and ## (*P* < 0.005) indicated significant difference at 7 days after HI.


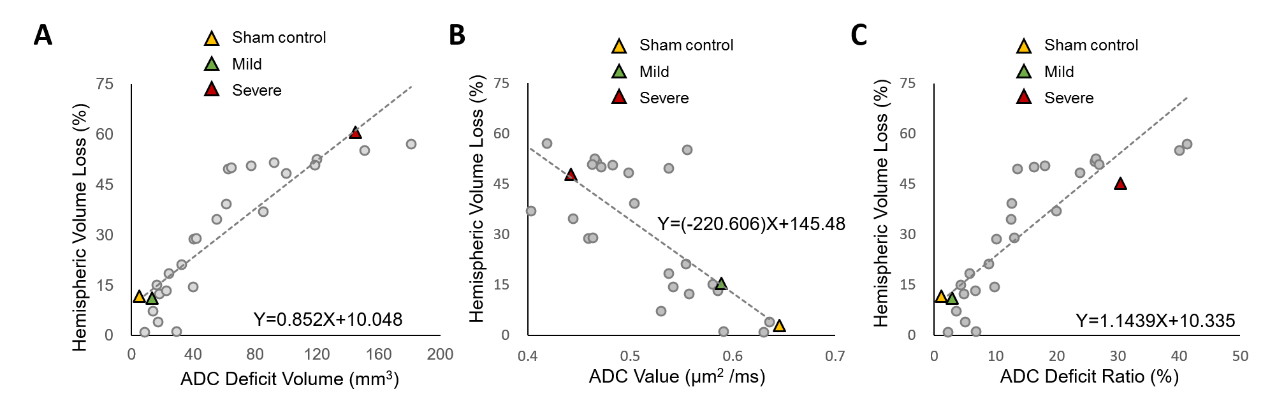


**Supplementary Figure 3** **The MR characteristics within 6 h after HI from the three representative animals for TEM.** The ADC-derived deficit volume (**A**), ADC values (**B**) and ADC derived deficit ratio (**C**) of the sham control, mild and severe pups shown in Figure 7 were denoted in the raster plot. The outcome severity of the animals for TEM were predicted based on the early changes of ADC-derived parameters.
